# Mild cognitive dysfunction in hereditary spastic paraplegia 4 disease related to fluorodesoxyglucose cerebral positron emission tomography

**DOI:** 10.1093/braincomms/fcaf382

**Published:** 2025-10-07

**Authors:** Raphaël Miroglio, Armand Hocquel, Jean-Marie Ravel, Guillemette Clément, Salomé Puisieux, Mylène Meyer, Laura Lavigne, Céline Dillier, Liang Liao, Emmanuelle Schmitt, Lucie Hopes, Maud Michaud, Sophie Pittion-Vouyovitch, Laetitia Lambert, Marine Guillaud-Bataille, Guillaume Banneau, Carine Bossenmeyer-Pourié, Céline Bonnet, Antoine Verger, Mathilde Renaud

**Affiliations:** Department of Neurology, CHRU Nancy, Nancy 54000, France; Department of Neurology, CHRU Nancy, Nancy 54000, France; Laboratory of Medical Genetics, CHRU Nancy, Nancy 54000, France; University of Lorraine, Inserm U1256, NGERE, Nancy F-54000, France; Department of Neurology, CHRU Nancy, Nancy 54000, France; Department of Neurology, CHRU Nancy, Nancy 54000, France; Department of Neurology, CHRU Nancy, Nancy 54000, France; Department of Neurology, CHRU Nancy, Nancy 54000, France; Department of Neurology, CHRU Nancy, Nancy 54000, France; Neuroradiology Department, CHRU Nancy, Nancy 54000, France; Neuroradiology Department, CHRU Nancy, Nancy 54000, France; Department of Neurology, CHRU Nancy, Nancy 54000, France; Department of Neurology, CHRU Nancy, Nancy 54000, France; Department of Neurology, CHRU Nancy, Nancy 54000, France; University of Lorraine, Inserm U1256, NGERE, Nancy F-54000, France; Department of Medical Genetics, CHRU de Nancy, Nancy 54000, France; Genetic Department, University of Sorbonne, AP-HP, GH Pitié-Salpêtrière, Paris 75013, France; Genetic Department, University of Sorbonne, AP-HP, GH Pitié-Salpêtrière, Paris 75013, France; University of Lorraine, Inserm U1256, NGERE, Nancy F-54000, France; Laboratory of Medical Genetics, CHRU Nancy, Nancy 54000, France; University of Lorraine, Inserm U1256, NGERE, Nancy F-54000, France; Department of Nuclear Medicine and Nancyclotep Imaging Platform, Université de Lorraine, CHRU; IADI, INSERM U1254, Nancy 54000, France; Department of Neurology, CHRU Nancy, Nancy 54000, France; University of Lorraine, Inserm U1256, NGERE, Nancy F-54000, France; Department of Medical Genetics, CHRU de Nancy, Nancy 54000, France

**Keywords:** SPG4, *SPAST*, 18F-FDG PET, frontotemporal, cognitive impairment

## Abstract

Autosomal dominant spastic paraplegia type 4 (SPG4, *SPAST* gene) is commonly described as a pure phenotype, with progressive spastic weakness of the lower limbs. Some cognitive disorders have been reported but remain difficult to characterize. Brain flurodesoxyglucose (18F-FDG) PET is a sensitive biomarker of glycolytic metabolism and shows loss of neuronal activity. Our objective was to describe the cognitive impairment of SPG4 patients, supported by brain 18F-FDG PET, to characterize the cognitive pattern and its localization. Twenty subjects from the Grand Est region, with a pathogenic variant in the *SPAST* gene, were included. Each patient had to undergo a neuropsychological assessment, a brain 18F-FDG PET scan, and a SPATAX-EuroSpa clinical assessment, including the Spastic Paraplegia Rating Scale (SPRS). Brain 18F-FDG PET was analyzed semiquantitatively after comparison with age and sex-matched control subjects. The study population was 65% (13/20) female, with an average age of 50 years (19–75 years). The median SPRS was 15/52 (12–45). Forty-six percent (7/15) of patients had a deficient Montreal Cognitive Assessment (MoCA) score (score <26) without any severe impairment (score <10). Assessments with a pathological score of <1.65 standard deviations or at the 5th percentile included verbal episodic memory in 16-item Free and Cued Recall Test (16-FCRT; 63%, 10/16), facial emotion recognition (56%, 9/16), and executive functions (Trail Making Test A and B; 47%, 7/15; the Stroop; 47%, 7/15; digit span of Weschler Adult Intelligence Scale 4; 38%, 6/16). Compared with those of control subjects, 18-FDG PET images of the brains of SPG4 subjects revealed frontotemporal and precuneus hypometabolism, principally in the prefrontal cortex, which was mainly mesial (*P* voxel value <0.001, corrected for the size of the familywise error (FWE) cluster, *k* = 1464). Frontal hypometabolism was correlated with age at onset (*k* = −0.475, *P* = 0.046) and the time to perform the Trail Making Test B test (*k* = −0.597, *P* = 0.019). Here, we describe a mild cognitive disorder clinically in SPG4 patients, associated with an hypometabolism on 18F-FDG PET imaging, which mainly corresponded to frontotemporal localization. Mild cognitive dysfunction should be screened in SPG4, as it can have a significant impact on socioprofessional life. Brain 18F-FDG PET, combined with neuropsychological assessment appears to be a good screening and follow-up examination for cognitive disorders.

## Teaser

Autosomal dominant spastic paraplegia type 4 (SPG4, *SPAST* gene) is commonly described as a pure phenotype with progressive spastic weakness of the lower limbs. Some cognitive disorders have been reported. Miroglio *et al*. show mild cognitive dysfunction in a cohort of 20 SPG4 patients with frontotemporal hypometabolism on fluorodesoxyglucose (18 F-FDG) PET.

## Graphical Abstract

**Graphical Abstract:**
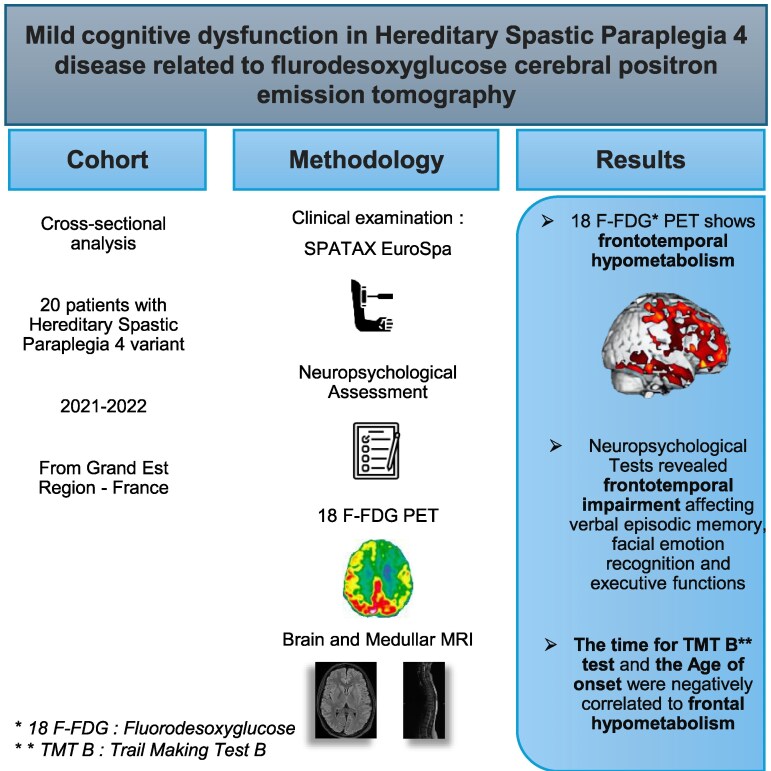

## Introduction

Hereditary spastic paraplegia (HSP) is an inherited neurodegenerative disease that causes progressive weakness of the lower limbs with a heterogeneous phenotype spectrum according to the gene involved, and spastic paraplegia 4 (SPG4) is the most common form of HSP.^1-3^ SPG4 is caused by a pathogenic variant in the *SPAST* gene at locus p22.3 of chromosome 2 and can be sporadic or inherited in an autosomal dominant manner. It is commonly acknowledged that SPG4-linked HSP is associated with pure phenotype, including spasticity and weakness in the lower limbs, pyramidal signs, urinary disturbances, and decreased vibration sense. However, various studies have reported mild or subclinical cognitive impairment, and some rare cases of dementia.^4-10^

Some studies have focused on functional brain imaging. Most nuclear imaging studies were performed with H_2_^15^O via PET,^11,12^ and three other studies have explored brain function with functional magnetic resonance imaging (fMRI).^13-15^

Unlike H_2_^15^O PET or perfusion scintigraphy, fluorodesoxyglucose (^18^F-FDG) PET is a nuclear imaging technique that allows the measurement of the cerebral metabolic rate of glucose (CMRGlc).^16^ CMRGlc and cerebral blood flow signals provide distinct information due to neurometabolic and neurovascular uncoupling.^17^ This CMRGlc is known to precede the relative transient decrease in blood-oxygen-level dependent (BOLD) signals used in fMRI without any magnetic limitations.^18^ Therefore, brain ^18^F-FDG PET is an early sensitive biomarker of neurodegeneration, routinely used in cognitive impairment, allowing the prediction of cognitive decline in patients without cognitive impairment, subjective cognitive impairment (SCI), or mild cognitive impairment (MCI).^16,19^ Moreover, all of the functional precedent imaging studies were undertaken with small sample size of SPG4 patients. In two fMRI studies, the population genotype was not precisely characterized or the study focused on motor function and cortex.^13,14^

Considering these gaps, we performed a study to elucidate a pattern of cognitive impairment in SPG4 patients supported by brain 18F-FDG PET.

## Population

We recruited patients from the Grand Est region in France between September 2021 and December 2022. Our inclusion criteria were as follows: an age >18 years and a pathogenic or likely pathogenic variant in *SPAST*.^20^ The exclusion criteria were an anterior or present diagnosis of another disease affecting cognition, a pathogenic variant in a gene associated to a HSP disease other than SPG4, and a contraindication for MRI or 18F-FDG PET. The institutional review board of the Centre Hospitalier Régional Universitaire de Nancy approved this study (2021PI148). All the participants provided written informed consent.

## Materials and methods

Participants were invited to undergo a brain 18F-FDG PET scan during a half day and, on another day, a clinical examination with an interview, a neuropsychological assessment, a brain MRI, and a medullar MRI. All these examinations were performed within 6 months.

### Clinical assessment

Clinical examinations employed a standardized assessment based on the SPATAX scale,^21^ comprising the disability stage quoted from 0 to 7 (a score of 7 corresponds to a state confined to bed) and the Spastic Paraplegia Rating Scale (SPRS) scored from 0 to 52,^22^ evaluating the clinical severity of the disease (a higher score indicates a more severe disease). To explore quality of life, we used the EuroQol-5 dimension-3 level (EQ-5D-3L), which comprises five dimensions divided into three levels of perceived problems (1: no problem, 2: some problems, 3: severe problems), and the EuroQoL visual analog scale (EQ-VAS), which ranges from 0 to 100 (100 is the best possible state of health).^23^ The Patient Health Questionnaire-9 (PHQ-9), which ranges from 0 to 27 (27 corresponds to a very poor quality of life).^24^ The Instrumental Activities of Daily Living (IADL) score was chosen for evaluating the cognitive impact of the disease on functional autonomy, scored from 1− to 8 (8 is the complete autonomy).^25^ All general and detailed clinical assessment are described in Supplementary material (Tables S1 and S2A and S3B).

### Neuropsychological assessment

Before evaluation, we asked patients for psychiatric comorbidities, particularly sign of depression, that could interfere with the different tests. The participants had to undertake a standardized cognitive assessment including (1) Montreal cognitive assessment (MoCA test), which evaluates global cognitive function (≥ 26/30: no neurocognitive impairment; 18–25/30: mild impairment; 10–17: moderate impairment; < 10: severe impairment)^26^ ; (2) 16-item Free and Cued Recall (16-FCRT or ‘RL/RI-16’ for the French version) for verbal episodic memory using 16 words to read and memorize, providing category cues in case of difficulty and then assessing encoding, retrieval, and storage^27^; (3) Revised Brief Visuospatial Memory Test (BVMT-R) for non-verbal and visuospatial episodic memory^28^; (4) Benton Judgment of Line Orientation Test (BJLO) for assessing visuospatial skills^29^; (5) ‘Test de dénomination orale d’image’ or ‘DO80’, a French test with 80 pictures to denominate for the evaluation of lexical access and visual gnosies^30^; (6) ‘Batterie brève d'évaluation des praxies gestuelles’ for assessing gestural praxis^31^; executive functions evaluated by (7) the Frontal Assessment Battery (FAB),^32^ (8) the Trail Making Test A and B (TMT A and B), which explore visual scanning, motor function and mental flexibility,^33^ (9) the Stroop Test (version of the Grefex) for inhibition capacity^34^ and (10) the digit span of the Weschler Adult Intelligence Scale fourth version (WAIS-4) for short term memory and verbal working memory^35^; (11) phonological verbal fluency (naming as many words that start with the letter *P* as possible in 2 min) and categorical semantic fluency (naming as many animals as possible in 2 min)^36^; and (12) the facial emotion recognition (FER) task of the mini-Social and Emotional Assessment (mini-SEA) to explore the emotion recognition aspect of social cognition.^37^ Compared with normative values, neuropsychological data were classified into two categories: < 1 standard deviation (SD) or <10th percentile (PC) for borderline performances and <1.65 SD or <5th percentile for pathological performances. For tests involving various subtests, the whole test was considered <1.65 SD or <5th percentile if almost one of the subtests failed (valuable for the Free Recall And Cued Test, the WAIS-4, the Stroop, TMT A and B and BVMT-R). The detailed neuropsychological data with quantitative values are provided in the Supplementary material (Table S3A and B). Pathological values were interpreted considering age and educational level according to the International Standard Classified of Education (ISCED) classification ranged from 0 to 8 (0 correspond to nursery school, 8 a Doctorat).

### Brain imaging

The brain 18F-FDG PET scans were obtained with a digital PET/CT (Vereos, Philips®) 30 min after injection of 2 MBq/kg 18F-FDG and after a resting-state period, with patients remaining for 15 min in one-bed acquisition. Before injection, all patients fasted for at least 6 h and had blood glucose levels < 10 mmol/L according to the European guidelines.^16^ All the PET images were reconstructed via iterative methods and corrected for scattering, randomness, and attenuation with a CT scan. Brain and spinal cord MRI examinations were performed to exclude that could affect clinical and cognitive assessments. MRI was conducted on a 1.5-T magnet (SIGNA system, GE Healthcare, USA) with the following acquisition parameters: axial diffusion-weighted imaging (3 mm slice thickness), 3D T1-weighted imaging-(BRAVO sequence), 3D FLAIR imaging, and posterior fossa coronal T2. Spinal cord MRI included: sagittal T1-weighted, sagittal T2-weighted, and axial T2-weighted sequences centred at the C3 and C4, vertebral levels. MRI was interpreted by Neuroradiologists of the Neuroradiology department of the CHRU of Nancy.

### Genetic data

We collected genetic information from medical patient records and classified the significance of the different variants according to the American College of Medical Genetics and Genomics (ACMG) variant classification (1: benign, 2: likely benign, 3: uncertain significance, 4: likely pathogenic, 5: pathogenic). All patients had a heterozygous pathogenic variant.

### Statistical analysis

The means with the minimum and maximum values were calculated if the variables followed a normal distribution; otherwise, the medians, 25th quartiles and 75th quartiles were calculated. For PET data analysis, semiquantitative analyses were performed with the statistical parametric mapping software SPM 12 (https://www.fil.ion.ucl.ac.uk/spm/software/spm12/). Comparisons with healthy sex and age-matched patients from the PACTEP population based on 65 healthy subjects^38^ (ClinicalTrials.gov Identifier: NCT03345290) were performed. After normalization of images to the Montreal National Institute (MNI) space and smoothing with an 8mm Gaussian filter, voxel-based group comparisons were performed using age and sex as covariates, intensity normalization to the pons -a robust structure for intensity normalization of PET images-^39^ due to the presence of elderly patients, and an inclusive grey-matter mask encompassing the brainstem and the cerebellum. The statistical threshold was set at a *P*-voxel value of 0.001, uncorrected, *P*-cluster value of 0.05 and familywise error (FWE) corrected. The identified clusters were reported according to the anatomical automatic labeling (AAL) atlas (https://www.gin.cnrs.fr/en/tools/aal/), and extraction of the mean values of the maximal significant cluster was performed with Marsbar (https://marsbar-toolbox.github.io/). Spearman correlations were performed between the mean metabolic values, age, age of onset, disability level, SPRS score, clinical evaluation, and neuropsychological test values. The variables correlated with hypometabolism, were represented by linear regression. Wilcoxon and Kruskal–Wallis analyses were performed for subgroup comparisons (truncating versus missense mutation, age <50 years or >50 years). A *P* value <0.05 was considered significant. The R® software was used for statistical analyses.

## Supplementary Material

fcaf382_Supplementary_Data

## Figures and Tables

**Figure 1:**
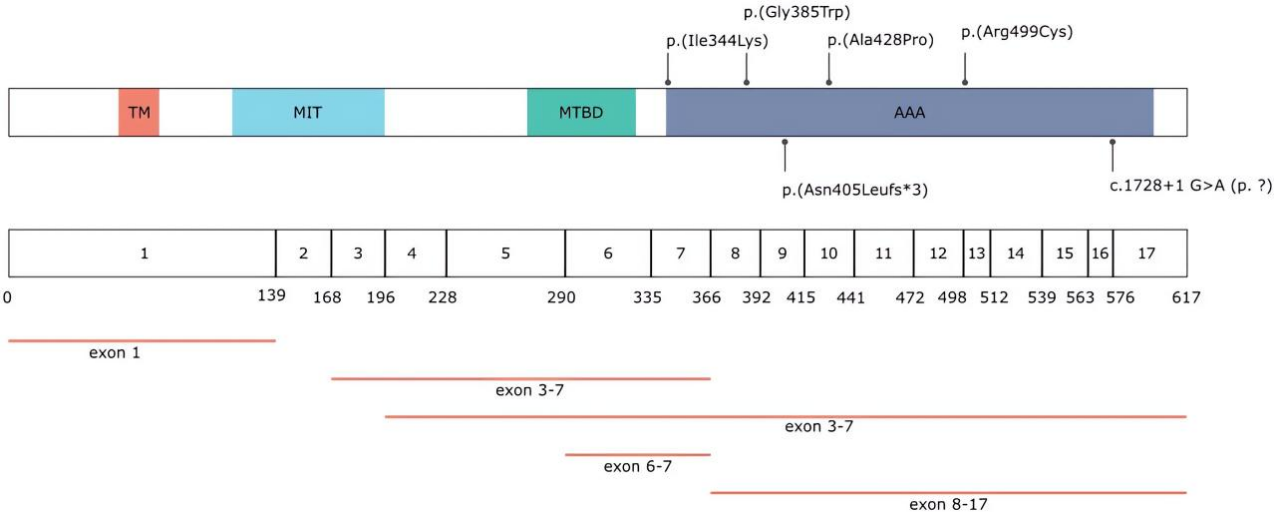
**Representation of the *SPAST* gene including SPG4 cohort variants.** Punctual SPG4 missense variants of the SPG4 cohort are indicated with plots of their corresponding localization on the gene, and large deletions are represented by lines with the concerning exon under. The different *SPAST* gene domains: AAA, ATPases associated with diverse cellular activity domains or ATPase cassettes; MIT, microtubule-interacting and trafficking domain; MTBD, microtubule-binding domain; TM, transmembrane domain.

**Table 1:** General data for the SPG4 cohort (*n* = 20)

	Male	Female		
Sex (*n* (%))	7 (35%)	13 (65%)		
	**All**			
Age (mean [min, max])	50 [19;75]			
Age at onset (mean [min, max])	33 [3;60]			
Disability stage (mean [min, max])	4 [1;6]			
SPRS (median [Q25;Q75])	15 [13;22]			
IADL (median [Q25;Q75])	8 [8;8]			
EQ-5D-3L (mean [min, max])	9 [5;12]			
EQ VAS (mean [min, max])	59 [35;90]			
PH9 (median [Q25;Q75])	4 [2;9]			
	**ISCED 2 or less**	**ISCED 3**	**ISCED 4 or more**	
Education level—ISCED scale	0 (0%)	20 (100%)	0 (0%)	
	**Deletion**	**Missense**	**Duplication**	**Splicing site**
Variant type (*n*, %))	9 (45%)	8 (40%)	2 (10%)	1(5%)

EQ VAS, Euroquol visual analog scale; EQ-5D-3L, EuroQOL-5D-3 level; IADL, Instrumental Activities of Daily Life; ISCED, International Standard Classification of Education from 0 (Early childhood education) to 8 (Doctorate or equivalent); PH9, Questionnaire de la santé du patient; Q25, 1st quartile; Q75, 3rd quartile; SPRS, Spastic Paraplegia Rating Scale.

**Table 2:** Genetic data for the different variants of the SPG4 cohort

Patient No.	Nucleotide change	Exon	Amino Acid change	Variant classification
1	exon del	Exon 4 to 17	-	Class 5
2, 5	c.1211_1212dup	Exon 9	p.Asn405Leufs*3	Class 5
3, 12, 13	exon del	Exon 8 to 17	-	Class 5
4	exon del	Exon 1	-	Class 5
6,18	c.1495C > T	Exon 13	p.Arg499Cys	Class 5
7	c.1728 + 1 G > A	Exon 16	p.?	Class 5
8	exon del	Exon 3 to 7	-	Class 5
9, 14, 16	c.1153G > T	Exon 8	p.Gly385Trp	Class 5
10, 20	c.1282G > C	Exon 10	p.Ala428Pro	Class 5
11, 15, 19	exon del	Exon 6 to 7	-	Class 5
17	c.1031T > A	Exon 7	p.Ile344Lys	Class 5

All patients were heterozygous (*n* = 20). del, deletion, dup, duplication.

**Figure 2:**
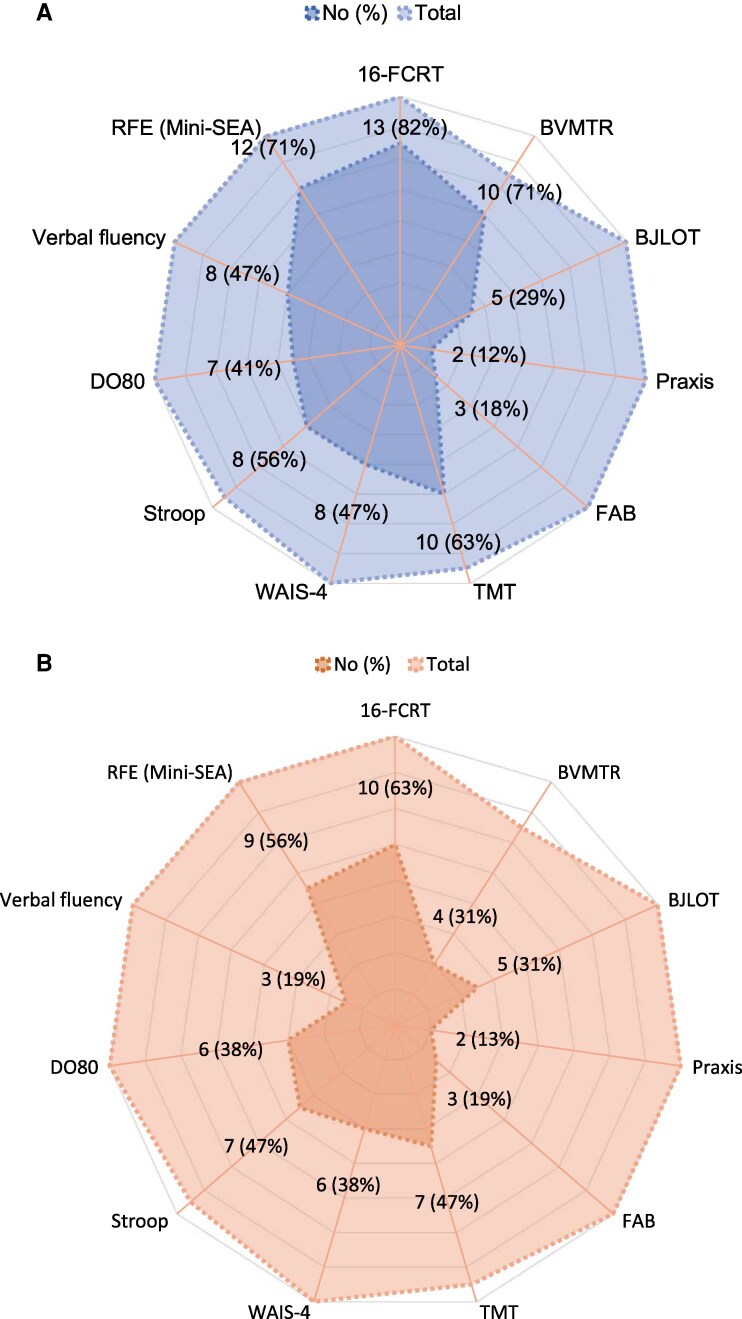
**Radar diagram resuming neuropsychological results of the SPG4 cohort.** The representation shows the proportion of patients (*No: number of patients with percentage of the population, all patients*  *=*  *17*) with values <1 SD or <10th percentile (A) or <1.65 SD or <5th percentile (B) for the different tests. 16-FCRT, 16 Free and Cued Recall Test; BJLOT, Benton Judgment of Line Orientation Test; BVMT-R, Brief Visuospatial Memory Test-Revised; DO80, ‘dénomination orale d’image’; FAB, Frontal Assessment Battery; Mini-SEA, mini-Social cognition & Emotional Assessment; RFE, Recognition of facial emotions; TMT, Trail Making; WAIS-4 (Digit-Span), Weschler Adult Intelligence Scale fourth.

**Table 3:** Results of the different neuropsychological tests of the standardized neuropsychological assessment (*n* = 16)

		*Median [Q25;Q75]*			
MocA		26 [23;28]			
	**Cognitive deficit**	***No/Total No (%)***			
	None (>25)	9/15 (60%)			
	Mild (18–25)	6/15 (40%)			
	Moderate (10–18)	1/15 (7%)			
	Severe (<10)	0/15 (0%)			
	**Score**	**>** **15, *No (%)***	**<15, *No (%)***		
FAB		13 (81%)	3 (19%)		
		**−1 SD or PC 10** ***No/Total No* (*%*)**	**−1 SD or PC 10** **Total of a test** ***No/Total No (%)***	**−1.65 SD or PC 5** ***No/Total No (%)***	**− 1.65 SD or PC 5** **Total of a test** ***No/Total No (%)***
16-FCRT	Immediate recall	6/16 (38%)	13/16 (81%)	5/16 (31%)	10/16 (63%)
	Free recall (FR1, FR2, FR3)	12/16 (75%)	-	8/16 (50%)	-
	Total recall	8/16 (50%)	-	6/16 (38%)	-
	Differed recall	9/16 (56%)	-	6/16 (38%)	-
	Recognition	4/16 (25%)	-	3/16 (19%)	-
BVMT-R	Immediate recall	6/13 (46%)	10/13 (77%)	4/13 (31%)	4/13 (31%)
	Differed recall	6/13 (46%)	-	4/13 (31%)	-
	Recognition	6/13 (46%)	-	0/13 (0%)	-
BJLO		5/16 (31%)	-	5/16 (31%)	-
DO80		7/16 (44%)	-	6/16 (38%)	-
Praxis		2/16 (13%)	-	2/16 (13%)	-
TMT	A	6/16 (38%)	10/15 (67%)	5/16 (31%)	7/15 (47%)
	B	8/15 (53%)	-	5/15 (33%)	-
WAIS4 Digit Span	Forward	3/16 (19%)	8/16 (50%)	2/16 (13%)	6/16 (38%)
	Backward	6/16 (38%)	-	4/16 (25%)	-
Stroop	Color	4/15 (27%)	8/15 (53%)	3/15 (20%)	7/15 (47%)
	Reading	6/15 (40%)	-	3/15 (20%)	-
	Interference	5/15 (33%)	-	5/15 (33%)	-
Verbal fluency		8/16 (50%)	-	3/16 (19%)	-
FER (Mini-SEA)		12/16 (75%)	-	9/16 (56%)	-

16-FCRT, 16 Free and Cued Recall Test; BJLOT, Benton Judgment of Line Orientation Test; BVMT-R, Brief Visuospatial Memory Test-Revised; DO80, ‘dénomination orale d’image’; FAB, Frontal Assessment Battery; FER, facial emotion recognition; Mini-SEA, mini-Social cognition & Emotional Assessment; PC 10, 10th percentile; PC 5, 5th percentile; Q25, 1st quartile; Q75, 3rd quartile; SD, standard deviation; TMT, Trail Making; WAIS-4, Weschler Adult Intelligence Scale fourth.

**Figure 3:**
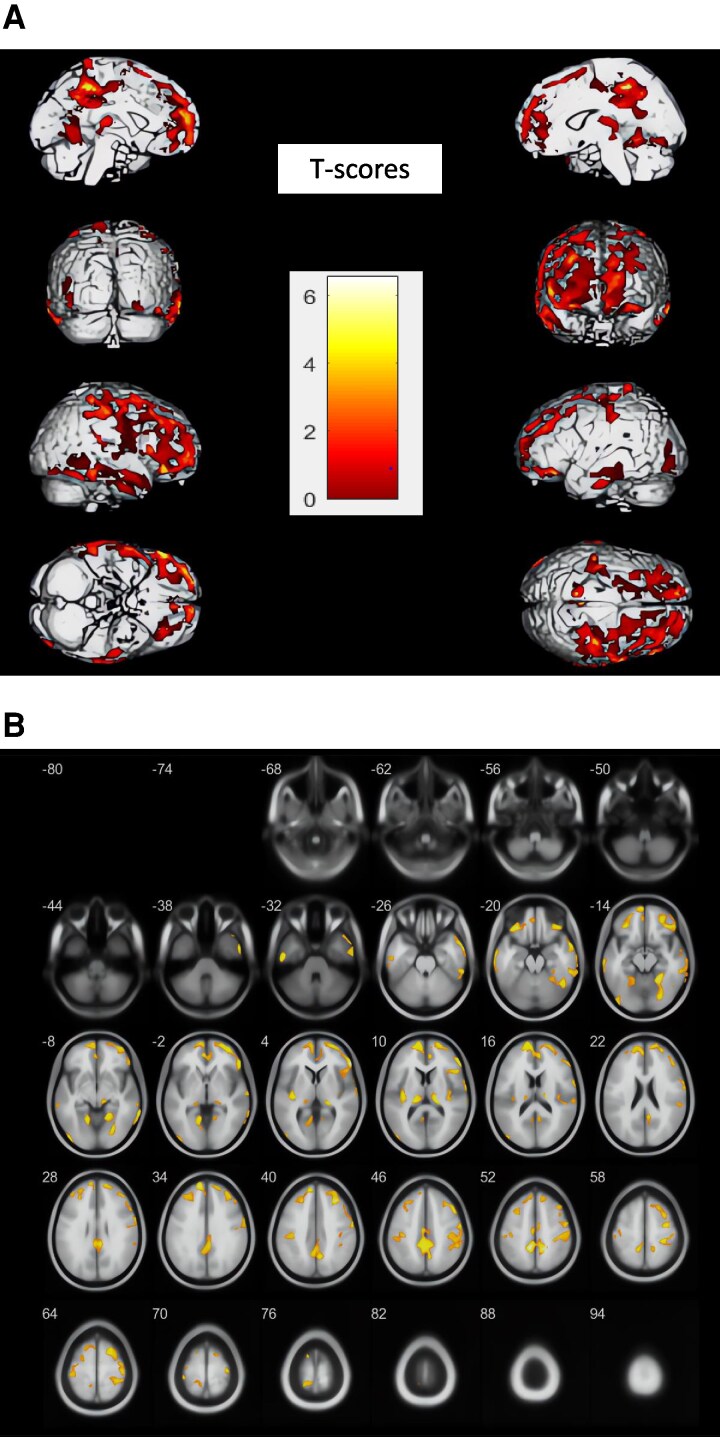
**3D and axial representations of 18 F-FDG PET scans of the SPG4 cohort.** Hypometabolic regions visualized on 3D representations (A) and axial representations projected onto MRI sections (B) of brain 18F-FDG PET scans. The observed hypometabolic regions are the bilateral prefrontal cortex, specifically the prefrontal mesial cortex, the superior prefrontal cortex, the precuneus and the temporo-occipital cortex (fusiform and lingual gyri). Student comparison of the SPG4 cohort (*n* = 20) and controls (*n* = 65), *P* voxel value <0.001, FWE corrected for the sample size, *k* = 1300.

**Figure 4:**
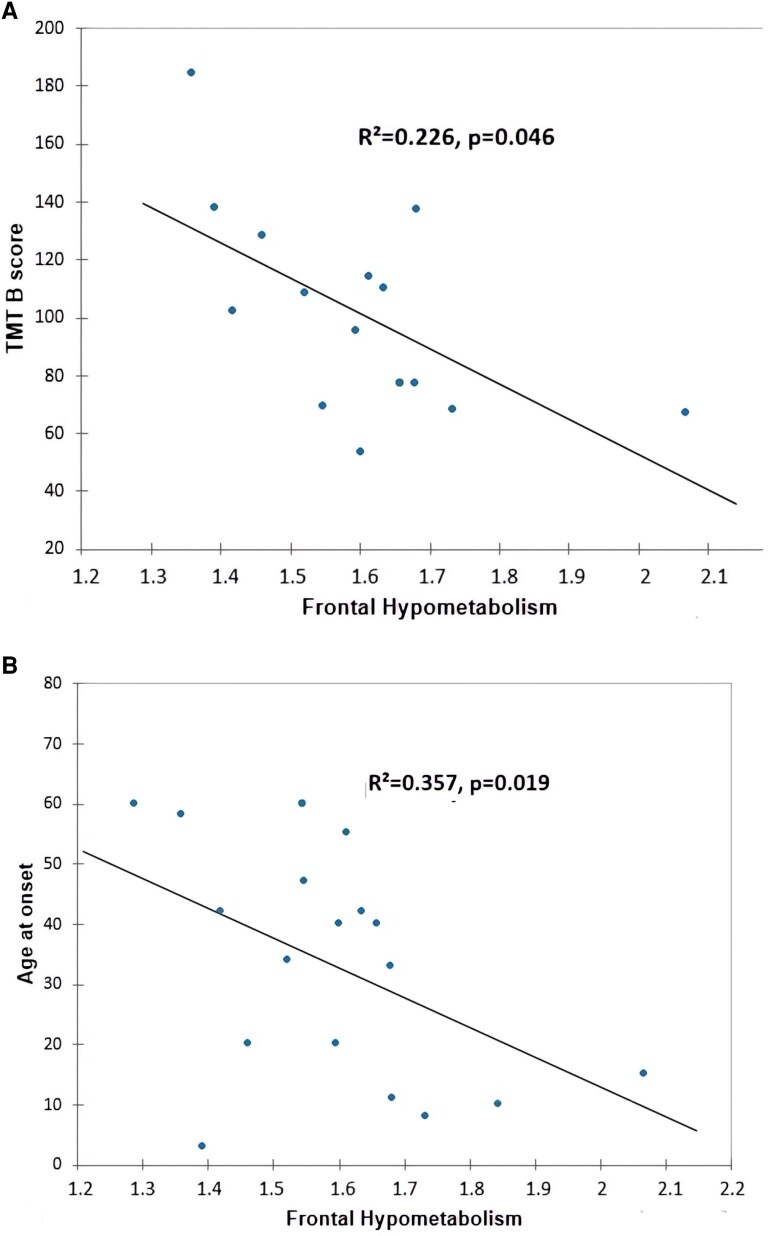
**Linear regressions between mean values of the maximal significant cluster of frontal metabolism and age at onset or TMT B score.** On *X*-axis, Age at onset is represented in years (**A**) (*k*  *=*  *−0.475, P*  *=*  *0.046*) and TMT B score in seconds (**B**) (*k*  *=*  *−0.597, P*  *=*  *0.019*). On *Y*-axis, mean values of the maximal significant cluster of frontal metabolism are represented.

## Data Availability

The authors confirm that the data supporting the findings of this study are available within the article or its Supplementary material.

## Results

### Population

Thirty patients were eligible for the analysis, among which 8 did not participate, one of whom was excluded for presenting with multiple sclerosis as a confounding disease. Subsequently, 22 patients were included, of whom one patient was excluded because of deregulated diabetes and one other because 18F-FDG PET could not be performed. From the 20 patients who underwent 18F-FDG PET and were included, 17 ultimately completed all the exams (brain 18F-FDG PET, brain and medullar MRI, neuropsychological assessment and clinical exam). Thus, brain 18F-FDG PET scans of 20 SPG4 patients (49.2 ± 15.8 years old, 62% women) were compared with those of 65 healthy control patients (49.6 ± 15.8 years old, 63% women).

### General data

The general data for the 20 patients who underwent 18F-FDG PET are presented in Table 1. The patients had a mean age of 50 years (19–75). The median SPRS was 15 (12− to 45). Missense *SPAST* variants were the most frequent type of variant (9/20, 45%), followed by intragenic deletions (9/20, 45%) and frameshifts (2/20, 10%). The different variants of the cohort are listed in Table 2 and represented on the *SPAST* gene in Fig. 1. With respect to autonomy, 79% of the patients had an IADL score of 8, one had a score of 3/8 (*data not shown*). One patient was placed in a sociomedical structure because of severe autonomy alteration and could not be evaluated for IADL. In terms of quality of life, the mean EQ-5D-3 L score was 9 and was correlated with the SPRS score (*Spearman correlation, P*  *=*  *0.0064, k*  *=*  *0.62*). The Kruskal–Wallis test revealed greater disability levels in patients with deletion and frameshift mutations than in those with missense mutations (*P*  *=*  *0.024, data not shown*). No ‘complex’ SPG4 phenotype was found in our cohort (*See*  Supplementary material Table S2A and B). Thus, we didn’t find motor affection that could interfere with the realization of some neuropsychological tests.

### Neuropsychological assessment

The MoCA test, described in Table 3, revealed alterations (<26) in 7/15 patients (44%), with one moderate impairment (<18). The numbers of patients with altered neuropsychological tests (classified between <1.65 SD or the 5th percentile and <1 SD or the 10th percentile) are presented in Table 3 and represented on radar diagrams in Fig. 2. With respect to executive functions, 3/16 patients (19%) had a pathological FAB score, and other scores were also well altered (<1.65 SD or 5th percentile): 7/15 patients (47%) in the Stroop test, 6/16 patients (38%) in the digit span of the WAIS-4 test, and 7/15 patients (47%) in the TMT A and B. For the other cognitive domains, 63% patients (11/16) had at least one of the 16-FCRT subtests <1.65 SD or the 5th percentile, with most difficulties experienced in one of the free-recall (50%, 8/16). A total of 56% (9/16) of the patients had clear limitations in the recognition of facial emotions, and 75% (12/16) had slight impairments (<1SD or 10 PC). Impairment of lexical access was also found in 38% of patients (6/16) with altered DO80. We found that the greater the importance of the SPRS, the longer was the duration for completing the TMT-B test (*Spearman correlation, k*  *=*  *0.62 P*  *=*  *0.0138, data not shown*). Other neuropsychological tests did not find significative correlations (*Spearman*) with SPRS, especially MoCA test. There was no statistical difference in MoCA Test results between the three mutation groups (*Kruskall-wallis, P*  *=*  *0.36*).

### Brain imaging

A comparison of the brain 18F-FDG PET scans of the SPG4 cohort (*n* = 20) and controls (*n* = 65) with Student comparisons (*P* voxel value <0.001), represented in Fig. 3, revealed hypometabolism in the bilateral prefrontal cortex, specifically the medial and superior prefrontal cortex, the precuneus and the temporo-occipital cortex (concerning the fusiform and lingual gyri). By analyzing general data with the maximal frontal hypometabolic cluster (*P* voxel values are described in table *S4* of *the* Supplementary material) via Spearman correlation, we detected negative correlations, represented by linear regression, between frontal metabolism and age at onset (*k*  *=*  *−0.475, P*  *=*  *0.046*, Fig. 4A) and between frontal metabolism and time to complete the TMT B test (*k*  *=*  *−0.597, P*  *=*  *0.019;* Fig. 4B). As expected, age was negatively correlated with hypometabolism (*Spearman correlation*, *k*  *=*  *−0.612 P*  *=*  *0.007, data not shown).* Brain MRI did not find significative atrophy, especially looking at frontotemporal, cerebellar, corpus callosum and thalamus atrophy. We exposed an example in Supplementary material (Fig. S1A and B) of a 76 years old patient with a MoCA score of 26/30 and moderate hypometabolism of the mesial prefrontal cortex associated to bilateral primary sensorimotor areas with predominant right-sided involvement.

## Discussion

The aim of this study was to better describe the cognitive impairment pattern in a large cohort of SPG4 patients subjected to metabolic imaging via brain 18F-FDG PET.

In summary, the neuropsychological assessment results revealed a frontotemporal deficit marked by important alterations predominating in verbal episodic memory, particularly for the retrievial, FER, some executive functions, i.e. inhibition processes, working memory, flexibility, processing speed and lexical access. The MoCA test, which is a screening and a non-exhaustive test for cognitive disorder, already showed an altered score in 46% (7/15) of the patients (Table 3). Recruiting patients aged at least 18 years, our cohort was heterogeneous in terms of age, highlighting that this cognitive impairment could also affect young patients for example, Patient No. 12, with a MoCA score of 18 at 26 years of age; Patient No. 14, with a FAB score of 14/18 at 34 years of age (*see*  Supplementary Material S3A). Nevertheless, this diffuse cognitive impairment was still mild with minimal impact on autonomy, although it could sometimes lead to a state of dementia. We described two elderly patients in an advanced state: one man aged 70 years with a MoCA test of 16/30 and an IADL of 7/8, and one woman aged 75 years who had been diagnosed with dementia supported by a Mini-mental Statement Examination (MMSE) score of 17/30 and lived in an accommodation for elderly dependent individuals. For this reason, she could not undergo a comprehensive neuropsychological assessment.

The group analysis of the ^18^F-FDG PET scans revealed bilateral hypometabolic regions, principally in the prefrontal cortex, specifically the medial and superior prefrontal cortex, and, to a lesser extent, in the precuneus and temporo-occipital cortex (fusiform and lingual gyri) (Fig. 3). Metabolic abnormalities in the prefrontal cortex could be linked to a high rate of difficulties in executive functions. Moreover, the impaired recognition of facial emotions (>50% of our patients) could be linked to the prefrontal mesial cortex.^40^ This alteration, which is part of the Mini-SEA test, has been previously reported by Scheuer *et al*.^11^ and Chamard *et al*.^10^ in a small cohort of SPG4 patients, as described in frontotemporal dementia (FTD).^37^ Nevertheless, the fusiform gyrus has been described as having a role in prosopagnosia^41^ and could be linked to the process of FER. This dysfunctional localization has been observed in PET marked with H_2_^15^O.^12^ A Chinese study used fMRI to ascertain the modification of the brain network in a resting-state in 12 males carrying the SPG4 variant.^15^ They reported decreased regional homogeneity in the bilateral superior frontal gyrus from the prefrontal cortex. Moreover, they reported decreased amplitude of low-frequency fluctuations (ALFF), a value that reflects spontaneous neuronal activity, in the left insular cortex, which has a role in cognitive functions and emotional processes. Thus, we confirmed and demonstrated an impairment in brain metabolic activity in our study, adding to this functional defect. However, it needs more studies to well understand the cognitive pattern of SPG4 disease.

We found, for the first time, a negative correlation, represented by linear regressions, between the hypometabolism of the frontal cortex and (1) the age of onset (*k*  *=*  *−0.475, P*  *=*  *0.046;* Fig. 4A) and (2) the time to perform the TMT B test (*k=*  *−0.597, P*  *=*  *0.019;* Fig. 4B). The first result is consistent with previous studies reporting that later onset provoked faster progression.^42,43^ Based on this results, we hypothesize that the *SPAST* variant leads to a mild neuronal deterioration in the brain beginning in the development phase, followed by a slow neurodegeneration compensated by the patient during an important part of life. After 40 years of age, there is a shift in which neuronal physiological senescence due to aging interacts with neuronal cell degeneration of SPG4 patients and potentiates the neurodegenerative process. Compared with that in controls, the blood level of neurofilaments in patients with the *SPAST* variant, reported by Kessler *et al*.,^44^ seems to increase around the age of 40 years, which supports our hypothesis. The Second result shows that more the frontal hypometabolism was low, more the time for doing the TMT B was long. This highlights the alteration of executive capacities due to loss of function of the frontal cortex. Moreover, we found that the greater the importance of the SPRS, the longer it took to complete the TMT B test (Spearman correlation, *k* = 0.62 *P* = 0.0138, data not shown). We can suspect that some cognitive tests, especially executive tests, could be correlated with the severity of the disease while there is no upper limb deficit.

The abnormalities observed in our study could be compared with the metabolic imaging pattern of FTD linked to amyotrophic lateral sclerosis (FTD-ALS)^45^ and, to some extent, to ALS^46^ and primitive lateral sclerosis (PLS).^47^ We hypothesize that lesions of the cortico-spinal tract could be common findings that provoke collateral frontotemporal deterioration through different neuronal interconnections but that physio-pathological processes are still different. Studies on zebrafish with the *SPAST* variant have demonstrated significant apoptosis in the central nervous system (CNS), with only 20% spinal motor neuron degeneration.^48^ These findings could support the idea of a more global degeneration that is not only restricted to the cortico-spinal tract. This is even more true that one study shows grey and white matter volume loss on brain MRI in SPG4.^49^

This study has several limitations. We used the term ‘Mild cognitive dysfunction’ in our study and not ‘Mild Cognitive Impairment’ because the *Diagnostic and Statistical Manual of Mental Disorders* (DSM-V) definition includes the notion of cognitive decline or cognitive complaints. Our cross-sectional design does not authorize us to talk about cognitive decline, which needs to be proven in a prospective study, and not all the patients had cognitive complaints. Taking this into account, we could only hypothesize about MCI (following DSM-V definition). The increased in Neurofilament observed by *Kessler and al*. and the worsening of neuropsychological tests related with age, could be consistent in our hypothesis.^44^ We had a limited number of participants, owing in part to the rarity of the disease, leading to a limited statistical power; however, it was still the largest cohort examined for brain 18F-FDG PET scans. Thus, caution must be exercised in terms of interpretations based on a small sample of the population. Moreover, we need to consider some limitations to the different neuropsychological tests for cognitive evaluation with the threshold effect. Another point is that the IADL assessment may not be the most optimized test for evaluating autonomy in HSP patients since the disease can provoke motor disability. Previous studies have shown that mild dementia in SPG4 disease, like asymptomatic dementia, has no impact on autonomy.^4,7^ The Amsterdam IADL assessment could be better adapted to evaluate personalized autonomy, which is more time-consuming but more specific and discriminative.^50^

In addition, PET imaging could be used in combination with blood sampling for neurofilament or peripheric lymphoblastoid cells^51^ as a prognostic marker, showing over extents of the evolution of the disease. Thus, allowing the response to a potential future treatment to be followed. In the study by Kessler *et al*.,^44^ the neurofilament blood level of patients with SPG4 was more important than that of healthy individuals, but it was not correlated with the SPRS.^44^ A very interesting study would be to evaluate the correlation between the neurofilament blood level with PET imaging and neurocognitive assessment.

In conclusion, our study revealed a mild frontotemporal cognitive impairment in patients with SPG4 corroborated by metabolic abnormalities on 18F-FDG PET scans, validating the findings of previous clinical and neuroimaging studies in the largest cohort assessed to date. In these patients, MCI is now well recognized and should be broadcasted and screened. For better care, brain 18F-FDG PET scans in clinical practice combined with neuropsychological assessment could be important and interesting for evaluating cognitive impairment that progresses slowly throughout life, which may have a marked impact on socioprofessional life, especially since it affects young patients. These tools could help clinicians characterize the daily difficulties experienced by SPG4 patients and offer them adapted and personalized cognitive rehabilitation (speech therapy, neuropsychological follow-up).
