# Supplementary material for: Mild cognitive dysfunction in hereditary spastic paraplegia 4 disease related to fluorodesoxyglucose cerebral positron emission tomography
Source: Brain Commun. 2025 Oct 7;7(6):fcaf382. doi: 10.1093/braincomms/fcaf382 (PMC12574705; doi:10.1093/braincomms/fcaf382)
Supplement: fcaf382_Supplementary_Data [file fcaf382_supplementary_data.docx]

**Supplementary Material**

| **Patient No.** | **Sex** | **Age (years)** | **Age of onset** | **Handicap level** | **SPRS** | **IADL** | **EQ-5D-3L** | **EQ VAS** | **PH9** |
| --- | --- | --- | --- | --- | --- | --- | --- | --- | --- |
| 1 | M | 55 | 34 | 3 | 13 | 8 | 7 | 70 | 6 |
| 2 | F | 64 | 60 | 5 | 14 | 8 | 8 | 50 | 3 |
| 3 | M | 46 | 40 | 4 | 21 | 8 | 10 | 55 | 6 |
| 4 | M | 70 | 60 | 5 | 22 | 7 | 7 | 55 | 3 |
| 5 | F | 66 | 11 | 3 | 14 | 8 | 8 | 50 | 10 |
| 6 | M | 40 | 20 | 3 | 21 | 8 | 7 | 80 | 0 |
| 7 | F | 44 | 42 | 5 | 15 | 8 | 10 | 80 | 17 |
| 8 | F | 59 | 20 | 6 | 42 | 7 | 10 | 60 | 2 |
| 9 | F | 45 | 8 | 1 | 6 | 8 | 5 | 90 | 1 |
| 10 | F | 39 | 33 | 4 | 16 | 8 | 10 | 70 | 13 |
| 11 | F | 50 | 42 | 3 | 15 | 8 | 9 | 50 | 3 |
| 12 | F | 26 | 10 | 6 | 45 | 3 | 8 | 50 | 3 |
| 13 | M | 53 | 47 | 4 | 14 | 8 | 8 | 60 | 2 |
| 14 | M | 34 | 3 | 3 | 26 | 7 | 11 | 40 | 11 |
| 15 | M | 75 | 58 | 5 | 22 | 8 | 10 | 50 | 4 |
| 16 | F | 19 | 15 | 2 | 10 | 8 | 8 | 70 | 4 |
| 17 | F | 60 | 55 | 4 | 24 | 8 | 12 | 35 | 20 |
| 18 | F | 43 | 40 | 3 | 8 | 8 | 6 | 40 | 1 |
| 19 | F | 27 | ND | 1 | 1 | 8 | ND | ND | ND |
| 20 | F | 75 | ND | 4 | 11 | ND | ND | ND | ND |

**Table S1: General data for the SPG4 cohort**

| **Patient No.** | **Spasticity** | | | **Reflex** | | | | | | | **Ankle clonus** | **Plantar reflex** | **Hoffman sign** |
| --- | --- | --- | --- | --- | --- | --- | --- | --- | --- | --- | --- | --- | --- |
|  | **UL** | **LL** | **Walk** | **Medio-clavicular** | | **Bicipital** | | **Patellar** | | **Ankle** |  |  |  |
| 1 | 0 | + | + | +++ | +++ | | +++ | | +++ | | 0 | ↑↑ | 0 |
| 2 | 0 | + | ++ | + | +++ | | +++ | | +++ | | + | ↓ | 0 |
| 3 | 0 | +++ | ++ | + | ++ | | +++ | | +++ | | 0 | ↑↑ | + |
| 4 | 0 | +++ | +++ | + | + | | +++ | | +++ | | 0 | ↑↑ | 0 |
| 5 | 0 | + | ++ | + | ++ | | + | | ++ | | 0 | 0 | 0 |
| 6 | 0 | +++ | +++ | + | ++ | | - | | - | | 0 | ↑↑ | 0 |
| 7 | 0 | + | + | + | ++ | | +++ | | +++ | | + | ↑↑ | 0 |
| 8 | 0 | +++ | +++ | + | +++ | | +++ | | +++ | | 0 | ↑↑ | + |
| 9 | 0 | + | + | + | +++ | | +++ | | +++ | | 0 | ↑↑ | + |
| 10 | 0 | + | + | + | ++ | | +++ | | +++ | | 0 | ↑↑ | + |
| 11 | 0 | ++ | + | 0 | +++ | | +++ | | +++ | | 0 | ↑↑ | + |
| 12 | 0 | +++ | +++ | + | ++ | | +++ | | + | | + | ↑↑ | + |
| 13 | 0 | + | ++ | + | + | | + | | + | | 0 | ↑↑ | 0 |
| 14 | 0 | +++ | +++ | + | - | | +++ | | +++ | | 0 | ↑↑ | 0 |
| 15 | 0 | +++ | +++ | ++ | +++ | | +++ | | +++ | | 0 | ↑↑ | + |
| 16 | 0 | + | + | 0 | +++ | | +++ | | +++ | | 0 | ↑↑ | 0 |
| 17 | 0 | ++ | +++ | + | ++ | | ++ | | + | | 0 | 0 | + |
| 18 | 0 | + | + | + | + | | ++ | | + | | 0 | ↑↑ | 0 |

**Table S2 A: Clinical data for the SPG4 cohort**

| **Motor Deficit (MRC scale)** | | | | **Muscle Wasting** | | | | **Vibration sense at Ankle** | **Sensory deficit** | **Sphincter disorder** | | | | | | |  |
| --- | --- | --- | --- | --- | --- | --- | --- | --- | --- | --- | --- | --- | --- | --- | --- | --- | --- |
| **PUL** | **DUL** | **PLL** | **DLL** | **PUL** | **DUL** | **PLL** | **DLL** |  |  | **Urgenturia** | **Incontinence** | | **Retention** | | **Sexual disturbance** | |  |
| + | 0 | + | + |  | 0 | + | + | ↓↓ | 0 | + | | 0 | | 0 | | 0 | |
| 0 | 0 | + | 0 | 0 | 0 | 0 | 0 | ᴓ | prick | 0 | | 0 | | + | | 0 | |
| 0 | 0 | 0 | ++ | 0 | 0 | + | 0 | ↓↓ | prick | 0 | | 0 | | + | | + | |
| 0 | 0 | + | + | 0 | 0 | 0 | 0 | ᴓ | 0 | + | | + | | + | | + | |
| 0 | 0 | 0 | 0 | + | + | 0 | 0 | ↓↓ | 0 | + | | 0 | | 0 | | 0 | |
| 0 | 0 | 0 | 0 | 0 | 0 | 0 | 0 | ↓↓↓ | 0 | 0 | | 0 | | 0 | | 0 | |
| 0 | 0 | 0 | 0 | + | + | + | + | ↓↓ | cold | 0 | | + | | 0 | | + | |
| 0 | 0 | + | + | 0 | 0 | + | + | ᴓ | 0 | 0 | | 0 | | 0 | | 0 | |
| 0 | 0 | + | 0 | 0 | 0 | 0 | 0 | ↓↓ | prick | + | | 0 | | 0 | | 0 | |
| 0 | 0 | + | + | 0 | 0 | + | 0 | ↓↓↓ | 0 | + | | 0 | | + | | 0 | |
| 0 | 0 | + | + | 0 | 0 | + | 0 | ↓ | prick and cold | 0 | | + | | 0 | | 0 | |
| 0 | 0 | +++ | 0 | 0 | 0 | + | + | ᴓ | cold and touch | 0 | | + | | 0 | | 0 | |
| 0 | 0 | + | + | 0 | 0 | + | + | ↓↓ | 0 | 0 | | 0 | | + | | 0 | |
| 0 | 0 | ++ | + | 0 | 0 | + | + | ↓↓ | 0 | + | | + | | 0 | | + | |
| 0 | 0 | + | 0 | 0 | 0 | + | + | 0 | 0 | + | | + | | 0 | | + | |
| 0 | 0 | 0 | 0 | 0 | 0 | + | + | ↓ | prick | 0 | | 0 | | 0 | | 0 | |
| 0 | 0 | + | ++ | + | + | + | + | ↓ | 0 | 0 | | 0 | | 0 | | 0 | |
| 0 | 0 | 0 | + | 0 | 0 | 0 | + | ↓↓ | 0 | 0 | | + | | 0 | | 0 | |

**Table S2 B: Clinical data for the SPG4 cohort**

| **Patient No.** | **Education level (ISCED)** | **Psychiatric comorbidities** | **MoCa** | **FAB** | **16-FCRT** | | | | |
| --- | --- | --- | --- | --- | --- | --- | --- | --- | --- |
|  |  |  |  |  | **Immediate recall** | **Total free recall (FR1, FR2, FR3)** | **Total recall**  **(TR1, TR2, TR3)** | **Delayed recall** | **Recognition** |
| 1 | 3 | 0 | 27 | 17 | 16 | **25*** (8, **7***,1) | **44*** (**13*,** 16, 15) | 26 | 16+1 |
| 3 | 3 | 0 | 28 | 17 | **13**** | 33 (9, 11, 13) | 46 (14, 16, 16) | 29 | 16+1 |
| 4 | 3 | ND | **16**** | ND | ND | ND | ND | ND | ND |
| 5 | 3 | depressive symptoms | **23**** | 15 | 15 | **18**** (8, **4**, 6****) | **39**(13, 12**,14**)** | **20**** | **14****+2 |
| 6 | 3 | 0 | **23**** | **13**** | 15 | **22**(5**, 7**, 10**)** | **44****(**13***, 16, **15**)** | **26**** | 16+0 |
| 7 | 3 | Addiction (alcohol), depression | 28 | 15 | 15 | 34 (11, 11, 12) | 47 (15,16,16) | 31 | 16+0 |
| 9 | 3 | 0 | 28 | 17 | 16 | 40 (12, 13, 15) | 48 (16, 16, 16) | 31 | 16+0 |
| 10 | 3 | 0 | 26 | **14**** | 16 | **25**(7*,9*,9**)** | 47 (15,16, 16) | **27*** | 16+0 |
| 11 | 3 | 0 | **25**** | 17 | 16 | 31 (10, **8****,13) | 46 (15, 15, 16) | 32 | 16+0 |
| 12 | 3 | 0 | **18**** | 15 | 16 | 33 (11, 11, **11***) | **45*** (15, **14***, 16) | **28*** | 16+0 |
| 13 | 3 | 0 | **23**** | 16 | **14**** | **22**** (**5****, 9, **8***) | **42*(11****, 16, 15) | **20**** | **13**+0** |
| 14 | 3 | depressive symptoms | ND | **14**** | **14**** | **27****(9,**8**,10****) | **44**** (15,**13****,16) | **28*** | **15***+0 |
| 15 | 3 | 0 | 26 | 17 | **13**** | **11** (4**, 4**, 3**)** | **39**** (**11****, 14, **14****) | **18**** | 16+0 |
| 16 | 3 | 0 | 27 | 17 | 16 | **31*(8*,11*,12*)** | 47 (15,16,16) | **27**** | 16+0 |
| 17 | 3 | suicide attempt, depression | **24**** | 17 | **14**** | **20 (7, 6**, 7**)** | **36**(11**,14*,11**)** | **20**** | **12****+1 |
| 18 | 3 | depressive symptoms | 30 | 0 | 15 | 31 (**8***, 11, 12) | 44 (13, 15, 16) | 29 | 16+0 |

**Table S3 A: Neuropsychological data of the SPG4 cohort,** **<10th percentile or -1SD, **<5th percentile or -1.65SD*

| **BVMT-R** | | | **BJLO** | **DO80** | **Praxis** | **TMT A** | **TMT B** | **WAIS4 – Digit Span** | | | **Stroop** | | | **Verbal fluency** | | **FER**  **(Mini-SEA)** |
| --- | --- | --- | --- | --- | --- | --- | --- | --- | --- | --- | --- | --- | --- | --- | --- | --- |
| **Immediate recall** | **Differed recall** | **Recognition** |  |  |  |  |  | **Forward** | | **Backward** | **Color** | **Reading** | **Interference** | **Verbal** | **Categorical** |  |
| **10**** | **3**** | 6+0 | 14 | 78 | 23 | 37 | 108 | 5 | **3**** | | 0 | 0 | 115 | 18 | 22 | **9.85**** |
| **20*** | 9 | **5+1*** | 11 | **74**** | 23 | 30 | 77 | 6 | 4 | | 68 | 47 | **170** (2EC+ 2ENC**)** | 25 | 31 | **10.3**** |
| ND | ND | ND | ND | ND | ND | ND | ND | ND | ND | | ND | ND | ND | ND | ND | ND |
| **5**** | **3**** | **5+1*** | **6**** | 79 | 20 | 51 | 137 | 5 | **2*** | | 72 | 52 | 173 | 14 | 22 | **9.9**** |
| 26 | 8 | **5*+0** | 11 | 79 | 23 | 36 | **128* (+1E*)** | **4**** | 3 | | 66 | **58*** | 106 | **14*** | 31 | **9.4**** |
| **13** | **6**** | **5*+0** | **10**** | **78*** | 23 | 32 | **102** (+2E**)** | 5 | 4 | | 62 | **49*** | **139* (7EC+4ENC**)** | **20*** | **27*** | 14.14 |
| 27 | 10 | **5*+0** | 13 | **76**** | 23 | 35 **(+1E**)** | 68 | 6 | 5 | | 58 | 46 | 100 | **13*** | 31 | **9.43**** |
| 26 | 10 | **5*+0** | 11 | **74**** | 23 | **49**** | 77 | 6 | **3*** | | **65*** | **56**** | **158**** | **13**** | **17**** | **11.1*** |
| ND | ND | ND | 14 | **77**** | 22 | 25 | **110*** | 6 | 5 | | 69 **(+1ENC**)** | 51 | 137 | 16 | 27 | 12.9 |
| **23*** | **9*** | 6+0 | **9**** | **73**** | 21 | **51*** | ND | 5 | **3**** | | 69 | **64*** | **157**** | 12 | **15**** | **11.1*** |
| 29 | 12 | 6+**1*** | 15 | **77**** | 23 | 36 | 69 | 5 | 4 | | 60 | 45 | 96 | 18 | **17**** | **11.1*** |
| ND | ND | ND | 13 | 80 | 23 | **56**** | **138**** | **4**** | 3 | | 62 | **53**** | **124**** | **17*** | **23*** | **11.6** |
| **9*** | **4*** | 5+1 | 11 | 77 | **21**** | 44 | 184 **(+2E*)** | 7 | **3**** | | 65 | 41 | 129 | 21 | 20 | **11.1*** |
| **22**** | **8**** | 6+0 | **10*** | 79 | 22 | 21 | 67 | 6 | **3**** | | 51 | 41 | 98 | 20 | 31 | **10.71*** |
| ND | ND | ND | **8**** | 75 | **22**** | 34 **(+1E**)** | 114 | 5 | 4 | | ND | ND | ND | 24 | 19 | 12 |
| 31 | 12 | ND | 13 | 80 | 22 | 30 **(+1E**)** | 53 | 6 | 4 | | 58 | **50**** | 106 | **20*** | **29*** | **9.9**** |

**Table S3 B: Neuropsychological data for the SPG4 cohort,** **<10th percentile or -1SD, **<5th percentile or -1.65SD.*

| **Patient No.** | **Frontal cortex voxel values** |
| --- | --- |
| 1 | 1,521324894 |
| 2 | 1,545349787 |
| 3 | 1,659406046 |
| 4 | 1,287023964 |
| 5 | 1,681299911 |
| 6 | 1,4615719 |
| 7 | 1,419268001 |
| 8 | 1,595929637 |
| 9 | 1,734511136 |
| 10 | 1,678998716 |
| 11 | 1,635523018 |
| 12 | 1,845360572 |
| 13 | 1,547156815 |
| 14 | 1,393187412 |
| 15 | 1,358877708 |
| 16 | 2,068399851 |
| 17 | 1,613403439 |
| 18 | 1,602237919 |
| 19 | 1,842784744 |
| 20 | 1,432840006 |

**Table S4: Voxel values obtained from the frontal cortex with brain 18F-FDG PET of the SPG4 cohort.**


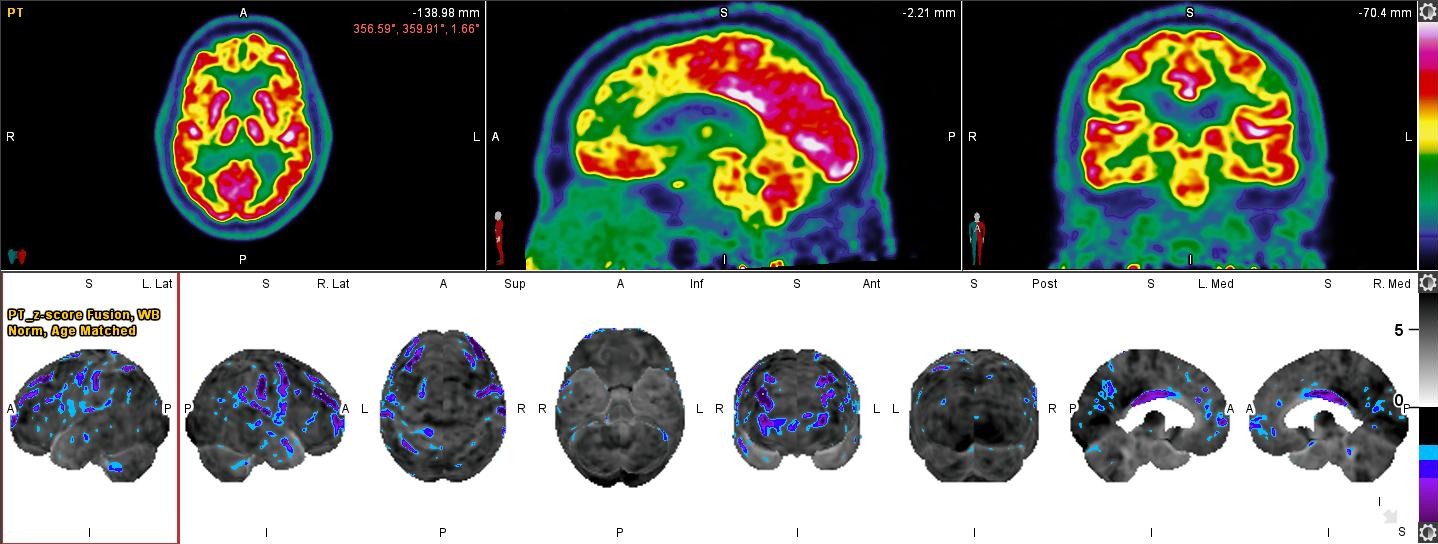


**Figure S1 A : 3D 18 F-FDG PET images representation of patient No. 16, a 75-year-old patient with SPG4 disease.First row, axial, sagittal and coronal slices. Second row : 3D volume representation with Z-scores of hypometabolism when matched with healthy controls (intensity normalization on the whole brain). He scored 26/30 on the MoCA test. He had limitations in work memory, difficulties in verbal episodic memory learning (spontaneous evocation and storage memory were impacted), and in nonverbal memory. He had a fragility in recognizing facial expressions, particularly those of sadness and fear. We found on 18 F-FDG PET a moderate hypometabolism of the mesial prefrontal cortex and bilateral primary sensorimotor areas with predominant right-sided involvement.**


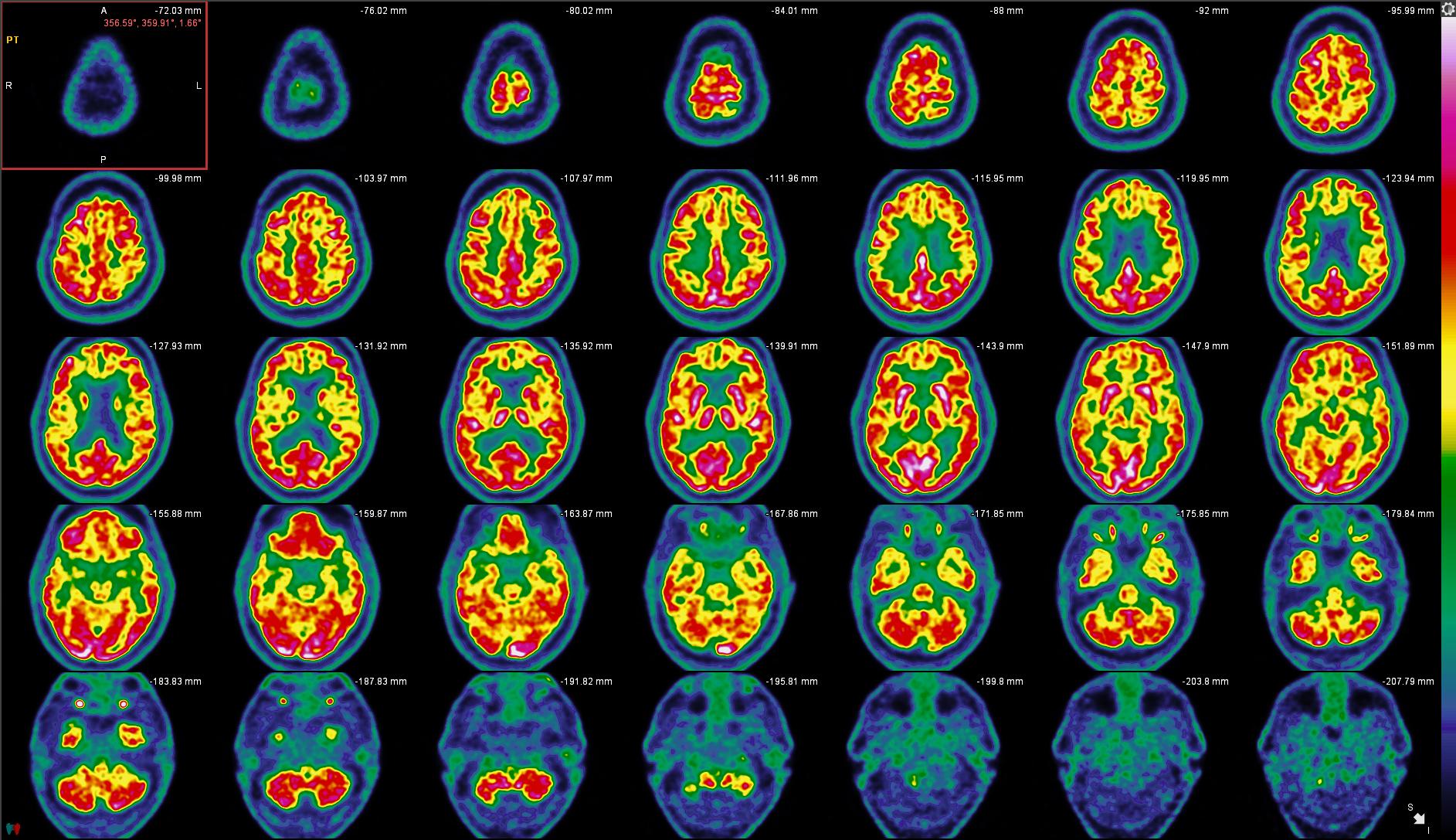


**Figure S1 B : Example of axial 18 F-FDG PET images of patient No. 16. A prefrontal mesial and sensory-motor areas hypometabolism is observed.**
